# Supplementary material for: Copine proteins are required for brassinosteroid signaling in maize and Arabidopsis
Source: Nat Commun. 2024 Mar 8;15:2028. doi: 10.1038/s41467-024-46289-6 (PMC10923931; doi:10.1038/s41467-024-46289-6)
Supplement: Supplementary file 1 — Supplementary Information [file 41467_2024_46289_MOESM1_ESM.pdf]

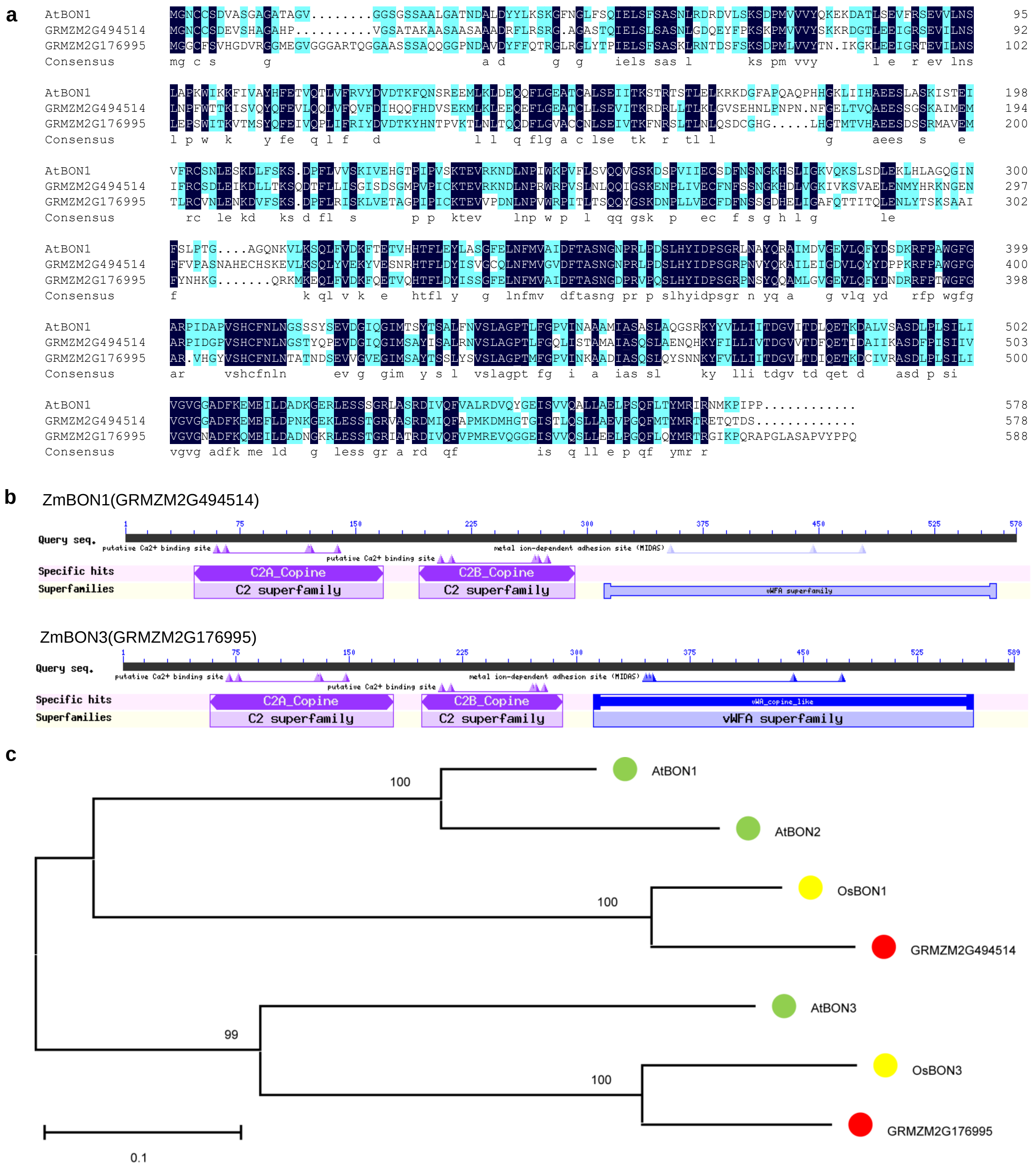

Supplementary Fig. 1|Prediction and characterization of BONZAI members in maize. a Alignment of the full-length amino acid sequences of AtBON1, ZmBON1 (encoded by GRMZM2G494514) and ZmBON3 (encoded by GRMZM2G176995) by DNAMAN. **b** Predicted domains of ZmBON1 and ZmBON3 by the online tools 'Conserved Domains' on NCBI (https://www.ncbi.nlm.nih.gov/Structure/cdd/wrpsb.cgi). Classical C2 domains at the N termini (C2A and C2B) and A domain at the C termini (vWFA) are shown. **c** Phylogenetic tree of BONZAI family proteins in Arabidopsis, rice and maize reconstructed using the neighbor-joining method with 1,000 bootstrap replicates in the MEGA7 software.

a

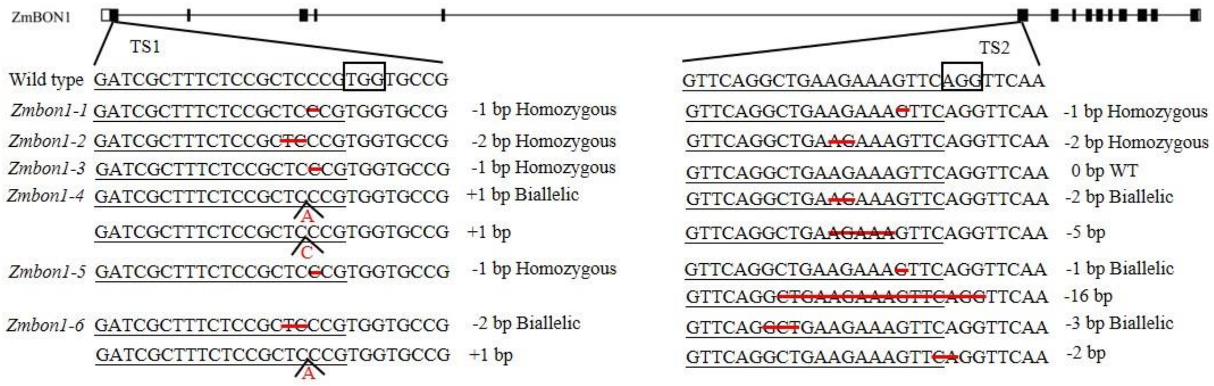

b

ID	Target and off-target sequence	Off-score	Gene	Region	Mutations
TS1	GATCGCTTTCTCCGCTCCCGTGG	1	GRMZM2G494514	CDS	-
Off-target-1	TAATGCTTTCTCCGCTCCCGTCG	0.061	GRMZM2G082207	intron	No
TS2	GTTCAGGCTGAAGAAAGTTCAGG	1	GRMZM2G494514	CDS	-
Off-target-1	GAGCAGGCTGAAGAAAATCCAGG	0.109	GRMZM5G852170	CDS	No
Off-target-2	GATCGTGCTGAAGAAAGTTCAAG	0.105	GRMZM2G064278	intron	No

c

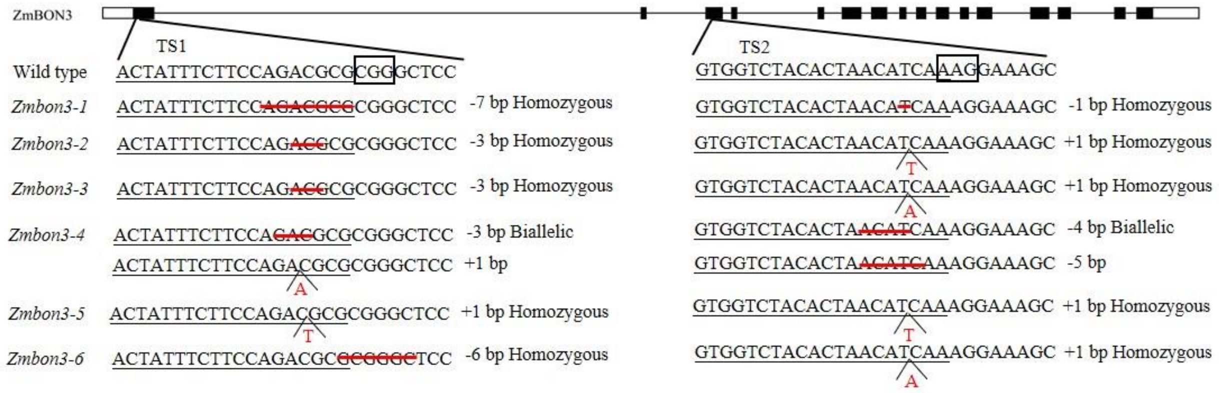

d

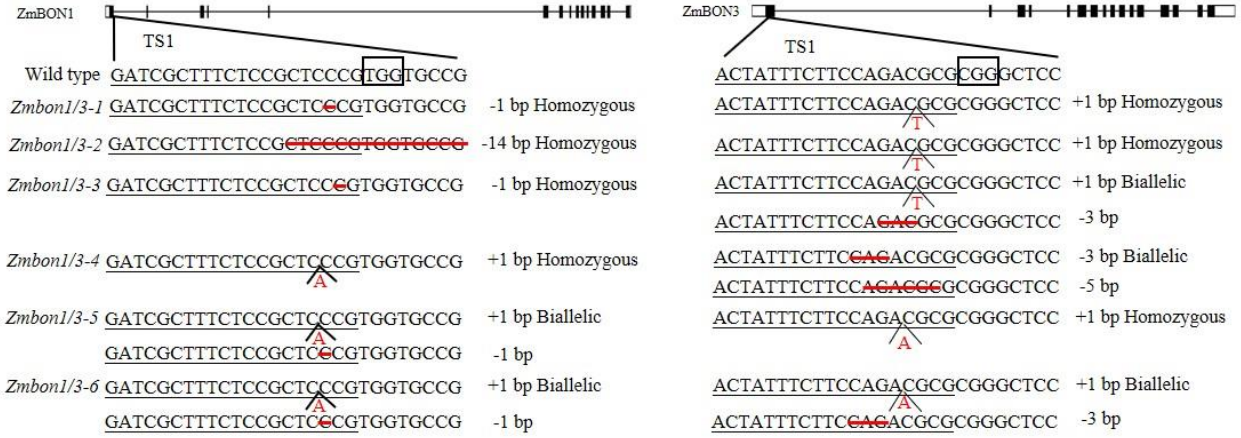

Supplementary Fig. 2|**Genotyping and off-target detection of *Zmbon1* and *Zmbon3* mutants. a**, **c**, **d** Genotyping of the different allelic mutants of *Zmbon1* (**a**) , *Zmbon3* (**c**), and *Zmbon1 Zmbon3* (*Zmbon1/3*) (**d**), generated by CRISPR/Cas9 gene editing. White boxes indicate 5' and 3' untranslated regions (UTRs), black boxes indicate exons, and black lines indicate introns. The 20-bp gene-specific target sequences and protospacer adjacent motif (PAM) are underlined and boxed, respectively. The number of nucleotides deleted and/or inserted is indicated by the minus (–) and plus (+) signs followed by a number. **b** Off-target detection of *Zmbon1*. No mutations were found in the three predicted off-target genes GRMZM2G082207, GRMZM5G852170, and GRMZM2G064278.

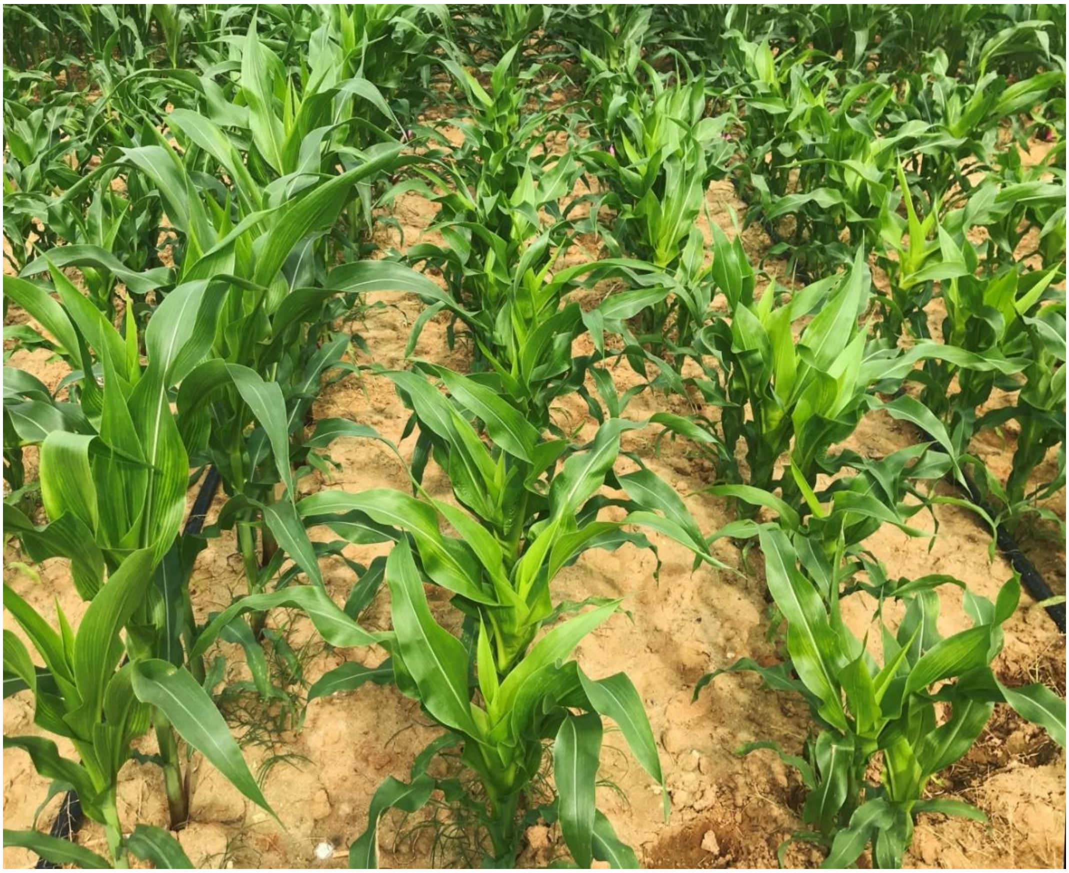

KN5585 *Zmbon1-1* *Zmbon1-2*

Supplementary Fig. 3|*Zmbon1* plants showed a dwarf phenotype in the field. Images of KN5585 and T2 plants of the *Zmbon1-1* and *Zmbon1-2* mutants planted in Sanya (109°17′ E, 18°35′ N) are shown.

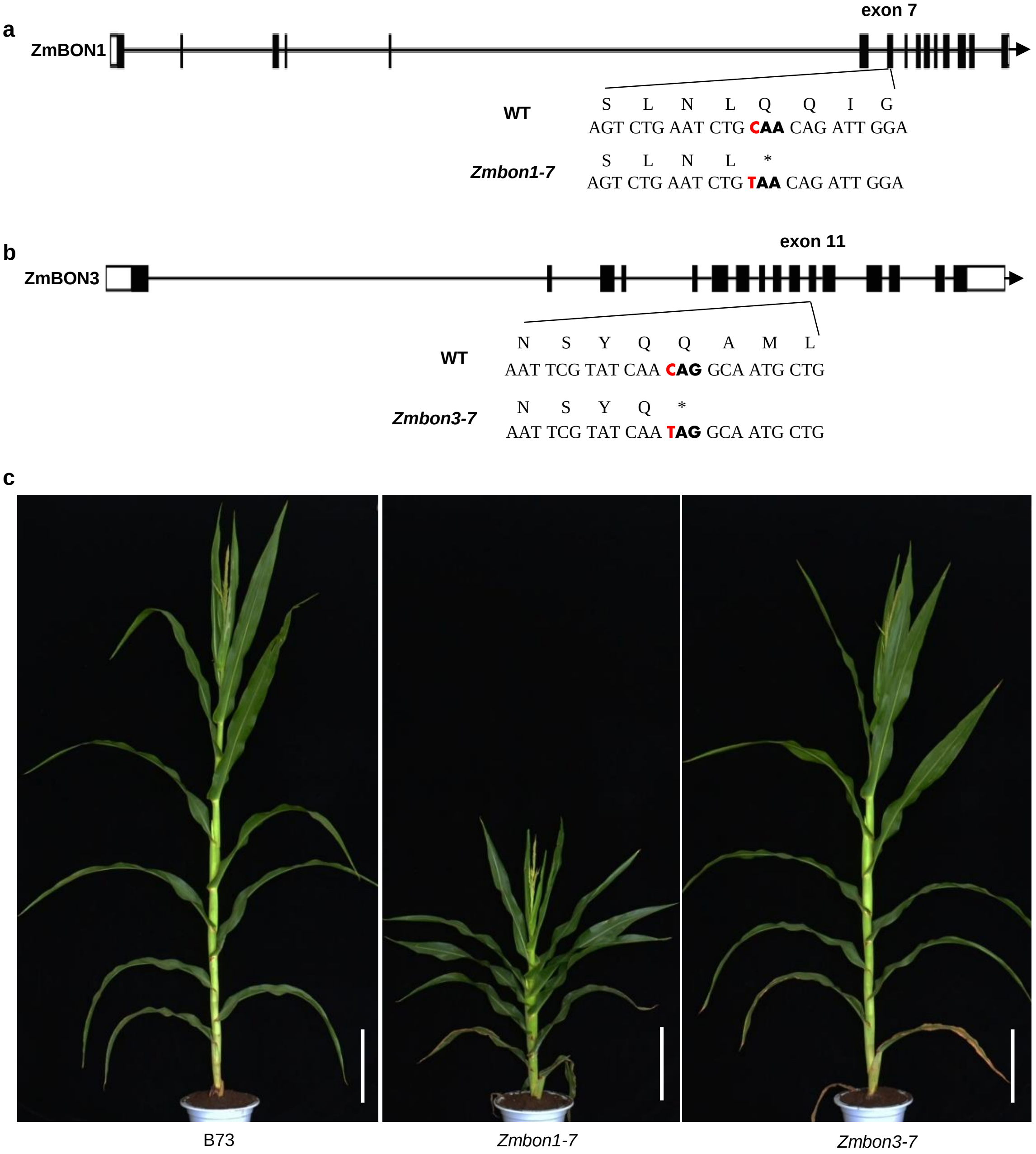

Supplementary Fig. 4|Genotype and morphology of *Zmbon1*-7 and *Zmbon3*-7 mutants in the B73 background. a, **b** Schematic diagram showing the mutation sites of *Zmbon1-7* (**a**) and *Zmbon3-7* (**b**). The C-to-T mutation in exon 7 of *ZmBON1* resulted in nonsynonymous substitution of the triplet codon CAA to TAA (**a**) and the C-to-T mutation in exon 11 of *ZmBON3* resulted in nonsynonymous substitution of the triplet codon CAG to TAG (**b**), leading to premature termination of translation. **c** Representative images of the wild-type B73, *Zmbon1-7* and *Zmbon3-7* grown in the field for 70 days. Scale bars, 20 cm.

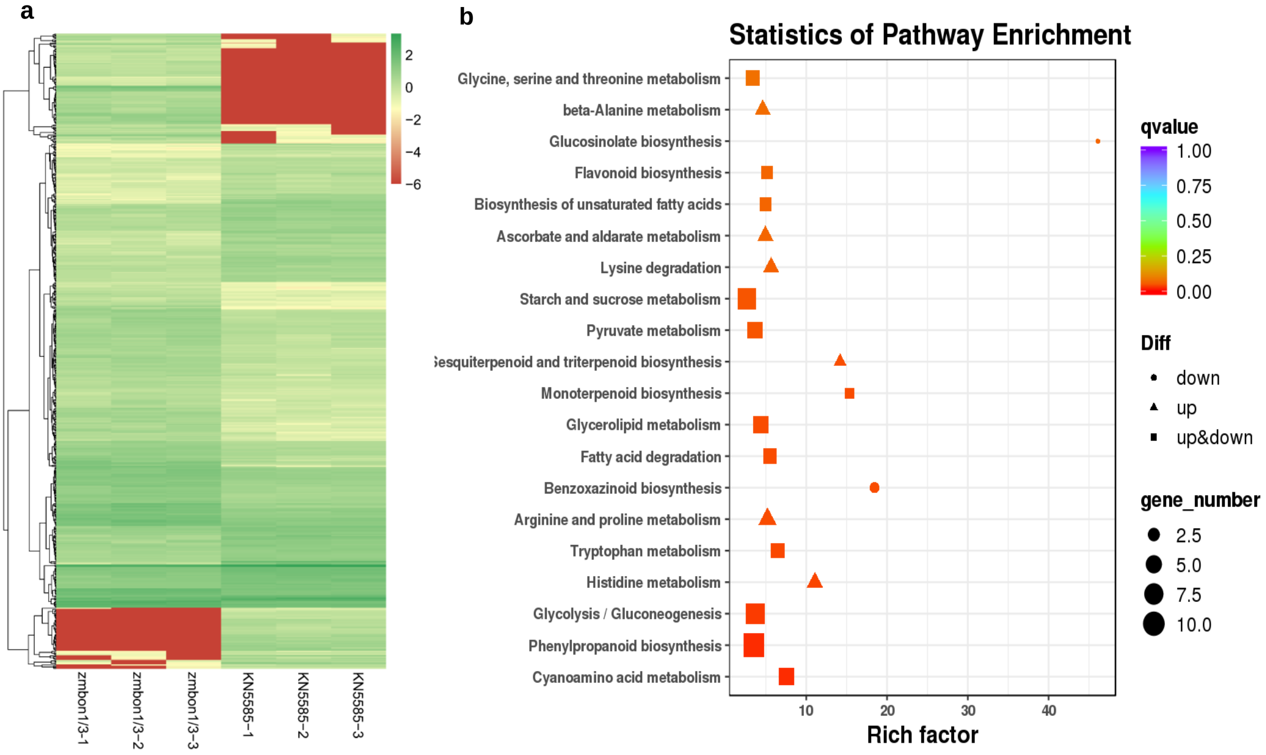

Supplementary Fig. 5|Differentially expressed genes in KN5585 vs *Zmbon1* from RNA-seq analysis. a Heatmap representation of hierarchical clustering of differentially expressed genes in KN5585 vs *Zmbon1/3*. **b** KEGG pathway enrichment analysis of differentially expressed genes in KN5585 vs *Zmbon1/3*. The top 20 most significant pathways are shown.

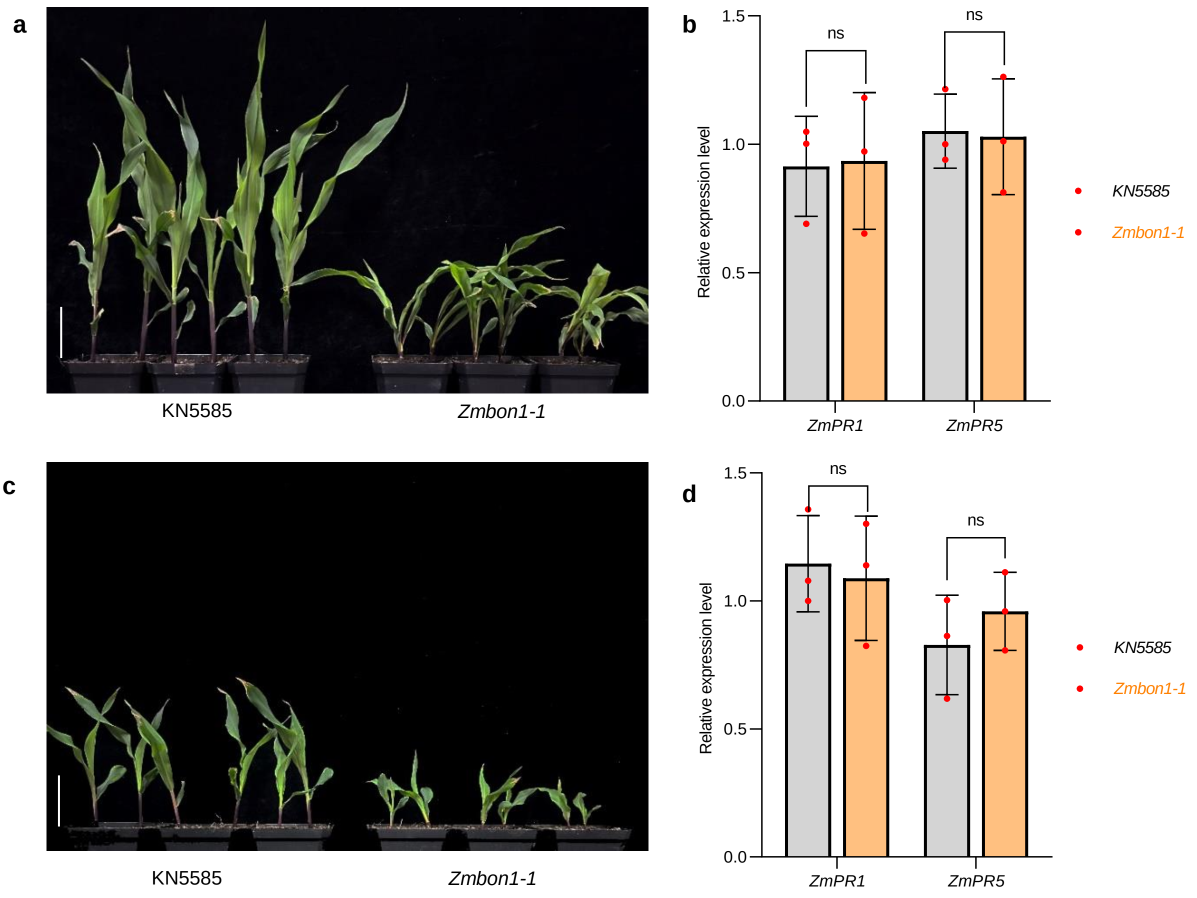

Supplementary Fig. 6|Characterization of the growth phenotype and defense-associated gene expression of *Zmbon1-1* at different growth stages. a, **b** Morphology and the relative transcriptional level of *ZmPR1/5* of KN5585 and *Zmbon1-1* at 4-leaf stage. **c**, **d** Morphology and the relative transcriptional level of *ZmPR1/5* of KN5585 and *Zmbon1-1* at 2-leaf stage. Scale bars, 5 cm. Significant differences were determined by two-tailed Student's *t*-tests at $P < 0.05$ (n = 3 biologically independent samples, ±SD) in b and d. Red dots represent individual data points. ns indicate no significant differences. Source data are provided as a Source Data file.

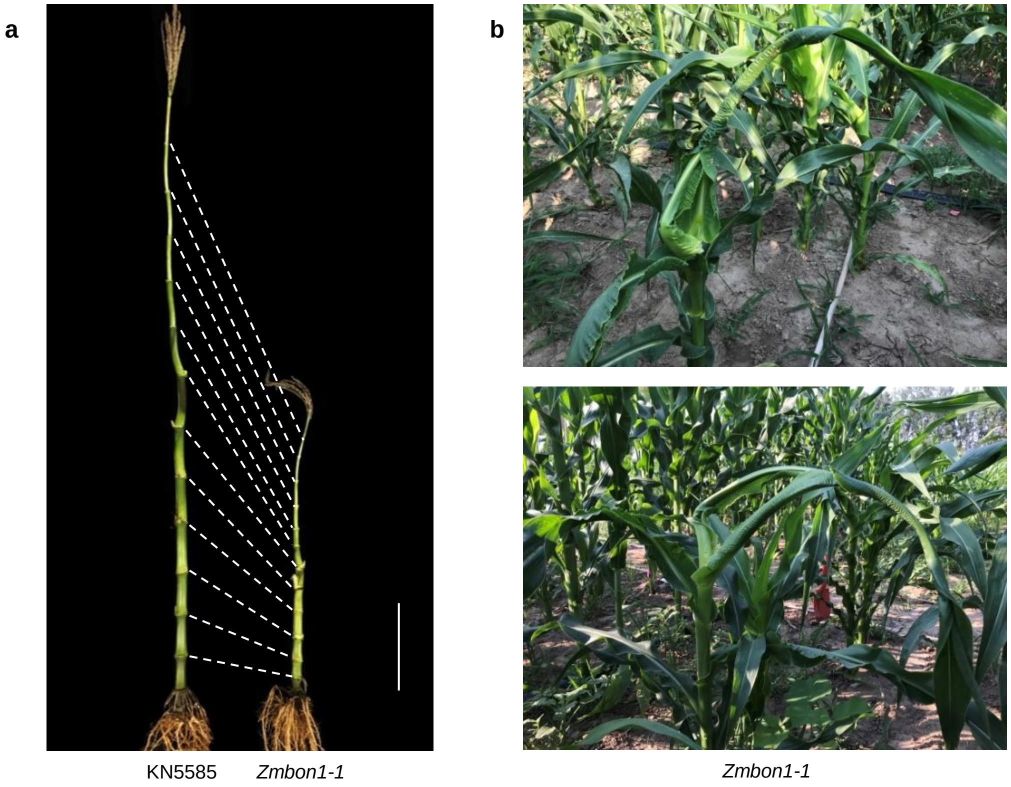

Supplementary Fig. 7|*Zmbon1-1* shows characteristics of BR-defective mutants. **a** Comparison of internodes between the wild-type KN5585 and *Zmbon1-1*. Scale bar, 20 cm. **b** Corkscrew appearance of *Zmbon1-1* plants.

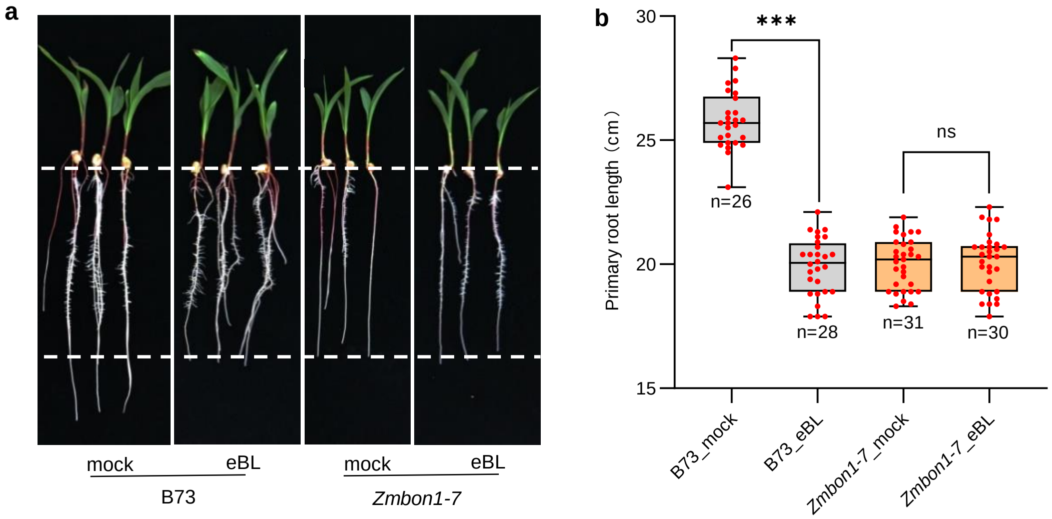

Supplementary Fig. 8|*Zmbon1-7* mutant plants are insensitive to BL treatment for primary root elongation. a Root elongation phenotypes of the wild-type B73 and *Zmbon1-7* seedlings under mock and eBL treatments. Scale bar, 5 cm. **b** Primary root length of B73 and *Zmbon1-7* mutant seedlings under a 100 nM eBL treatment. Data are displayed as box and whisker plots with individual data points. The whiskers represent maximum and minimum values, the center line represents the median and the box limits are the 25th and 75th percentiles. Ret dots represent individual data points. Different letters indicate significant difference between genotypes, which were determined by two-tailed Student's *t*-tests (***P < 0.001, ns represents P > 0.05). Source data are provided as a Source Data file.

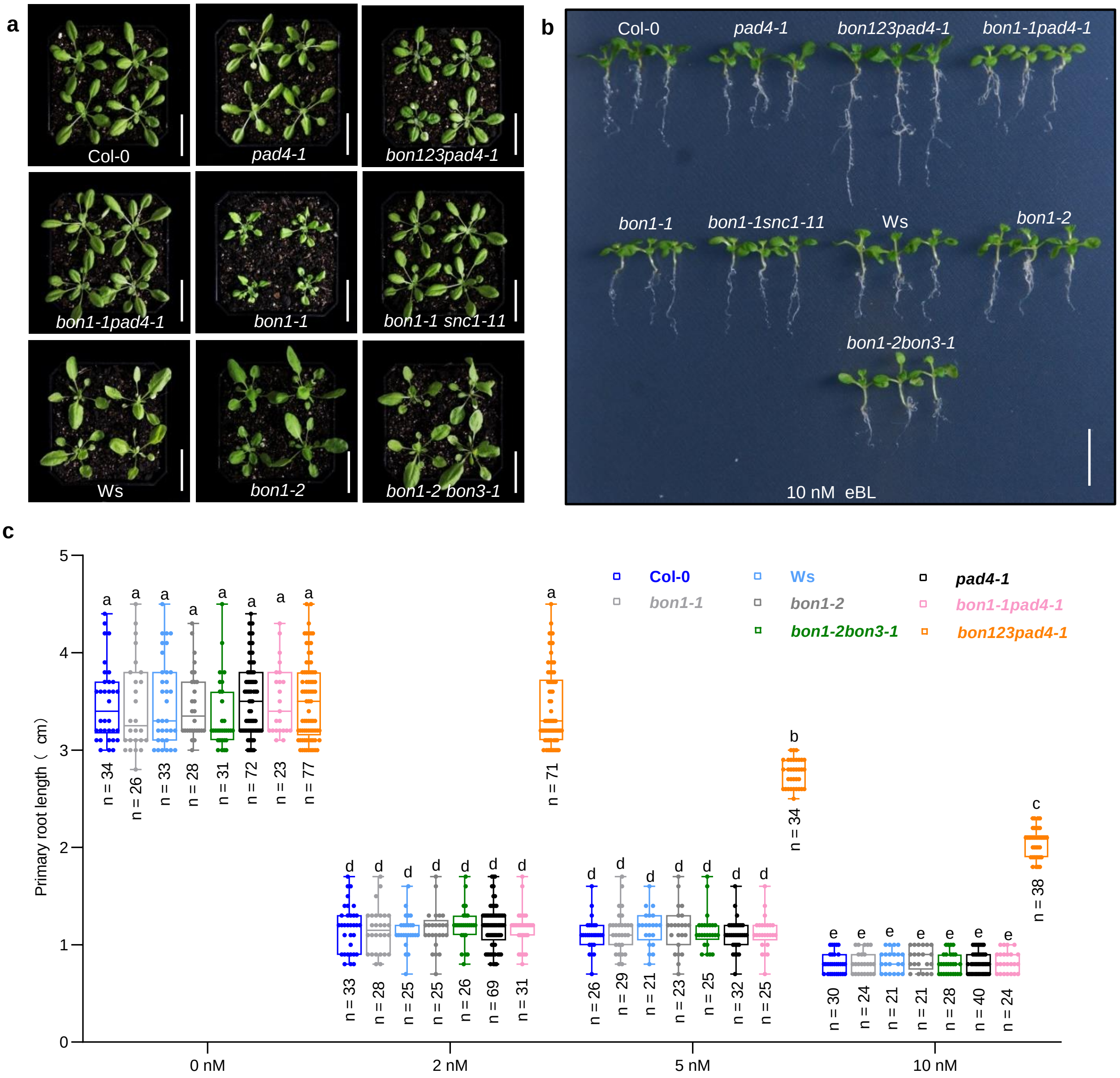

Supplementary Fig. 9|Phenotypes of Arabidopsis *BON* family mutants. a Plant morphology of soil-grown three-week-old Col-0 and Ws and *AtBON* family mutants in the Col-0 and Ws backgrounds. **b** Phenotypes of *AtBON* family mutants and associated wild types after 10 nM eBL treatment for 12 days on half-strength MS plates. **c** Primary root length of *BON* family mutants and wild types under treatment with 2, 5 or 10 nM eBL. Data are displayed as box and whisker plots with individual data points. The whiskers represent maximum and minimum values, the center line represents the median and the box limits are the 25th and 75th percentiles. Colored dots represent individual data points. Different letters indicate significant difference between genotypes, which were determined by one-way ANOVA at $P < 0.01$. Source data are provided as a Source Data file.

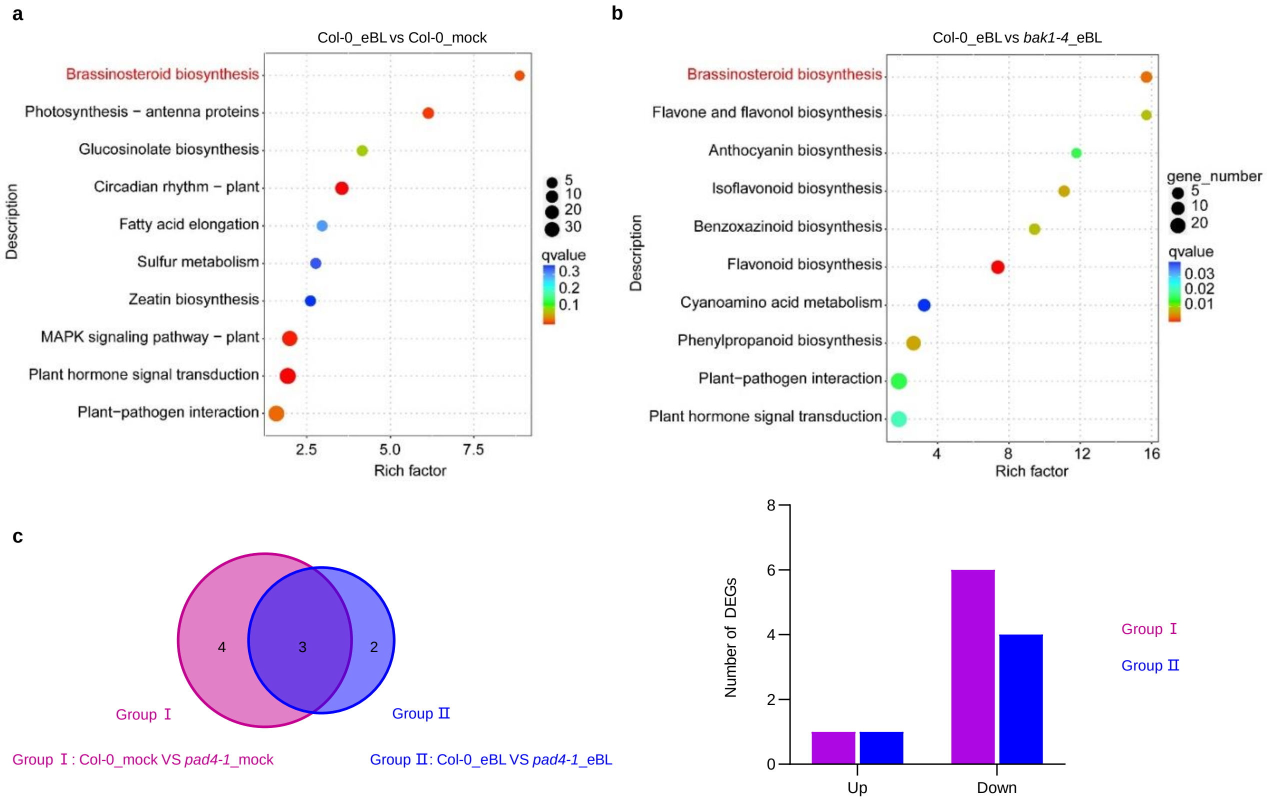

Supplementary Fig. 10|RNA-seq analysis of Col-0 and *pad4-1* seedlings after mock and eBL treatments. a, **b** Bubble chart of KEGG enrichment in DEGs in Col-0_eBL vs Col-0_mock (**a**) or Col-0_eBL vs *bak1-4*_eBL (**b**). Each dot represents a KEGG pathway. Y-axis, pathway; X-axis: enrichment factor. A larger enrichment factor indicates a more significant enrichment of the pathway. The color of the dots indicates the *q*-value. The size of the dots represents the number of DEGs enriched in each pathway. **c** Venn diagram showing the number of DEGs in Col-0_mock vs *pad4-1*_mock (Group Ⅰ) and Col-0_eBL vs *pad4-1*_eBL (Group Ⅱ). Source data are provided as a Source Data file.

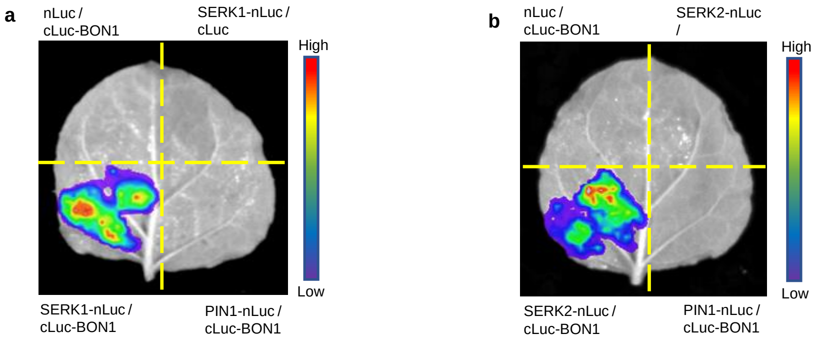

Supplementary Fig. 11 | Split luciferase complementation (SLC) assay of BON1 and BR signaling components. **a**, **b** SLC assay showing the interaction between BON1 and SERK1 (**a**) or SERK2 (**b**) *in vivo*. PIN1 was used as a negative control. All experiments were repeated 3 times with similar results.

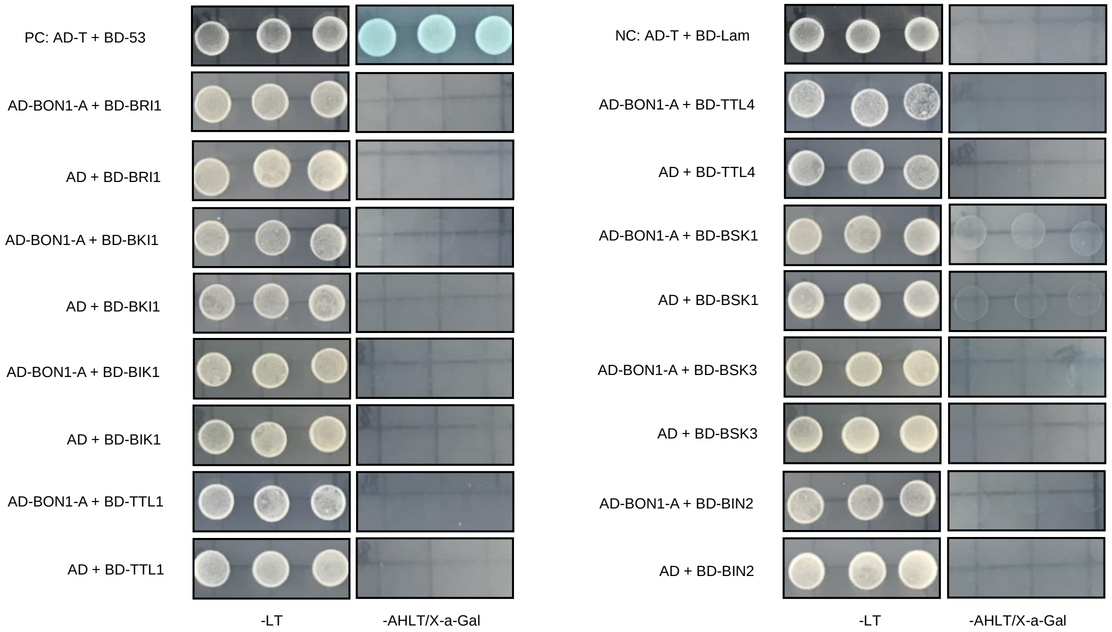

Supplementary Fig. 12|Y2H assay of interactions between the A domain of BON1 and BRI1, BKI1, BIK1, TTL1, TTL4, BSK1, BSK3 and BIN2. Constructs were co-transformed into yeast cells and grown on selective dropout medium as indicated. PC represents the positive control, NC represents the negative control.

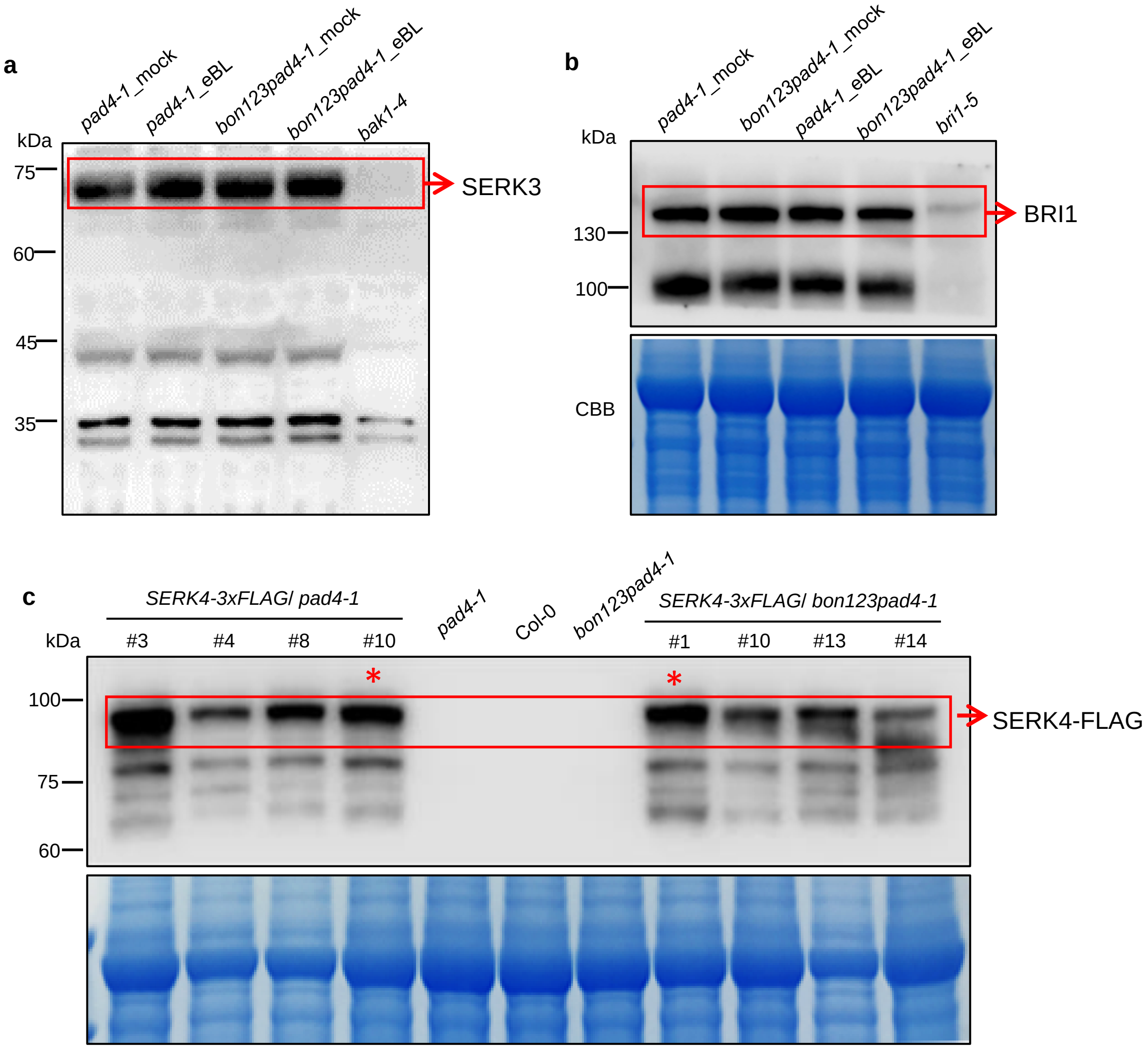

Supplementary Fig. 13|Validation of antibody specificity in the respective mutant and transgenic plants. a Immunoblot analysis of proteins extracted from mock- or eBL-treated *pad4-1*, *bon1-1 bon2-2 bon3-3 pad4-1* (*bon123pad4-1*) and *bak1-4* seedlings using anti-SERK3 antibody. **b** Immunoblot analysis of proteins extracted from mock- or eBL-treated *pad4-1*, *bon1-1 bon2-2 bon3-3 pad4-1* (*bon123pad4-1*) and *bir1-5* seedlings using anti-BRI1 antibody. **c**, Immunoblot of proteins extracted from Col-0, *pad4-1* and *bon1-1 bon2-2 bon3-3 pad4-1* (*bon123pad4-1*) seedlings and from different *SERK4-3xFLAG/pad4-1* and *SERK4-3xFLAG/bon123pad4-1* transgenic lines using anti-FLAG antibodies. Line 10 of *SERK4-3xFLAG/pad4-1* and line 1 *of SERK4-3xFLAG/bon123pad4-1* showed similar protein abundance and were used for the co-IP assay in Fig. 6**a**, **b**.

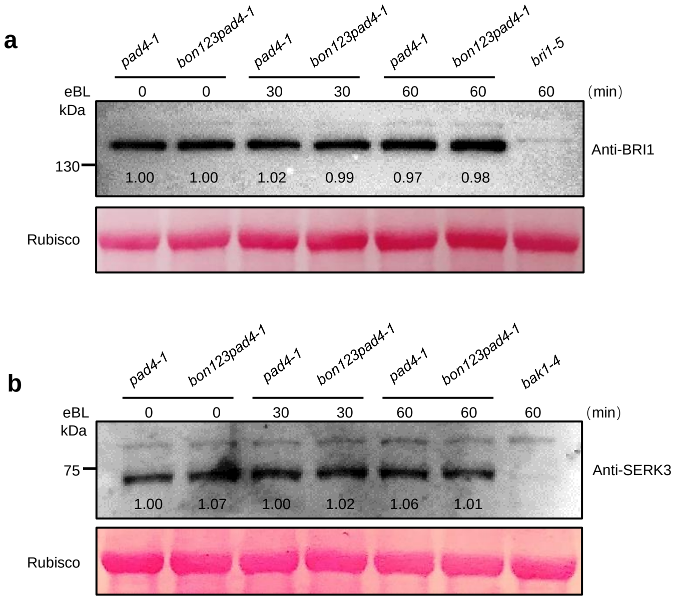

Supplementary Fig. 14|The abundance of BRI1 and SERK3 before and after eBL treatment. a, **b** the protein abundance of BRI1 (**a**) or SERK3 (**b**) in *pad4-1* with that in *bon1-1 bon2-2 bon3-3 pad4-1* (*bon123pad4-1*) seedlings subjected to eBL treatment for 0, 30 and 60 min. Ponceau S-stained Rubisco was used as a loading control. Numbers underlying each band denote their relative intensity normalized with the corresponding Rubisco band by ImageJ quantification. All experiments were repeated 3 times biologically.

.

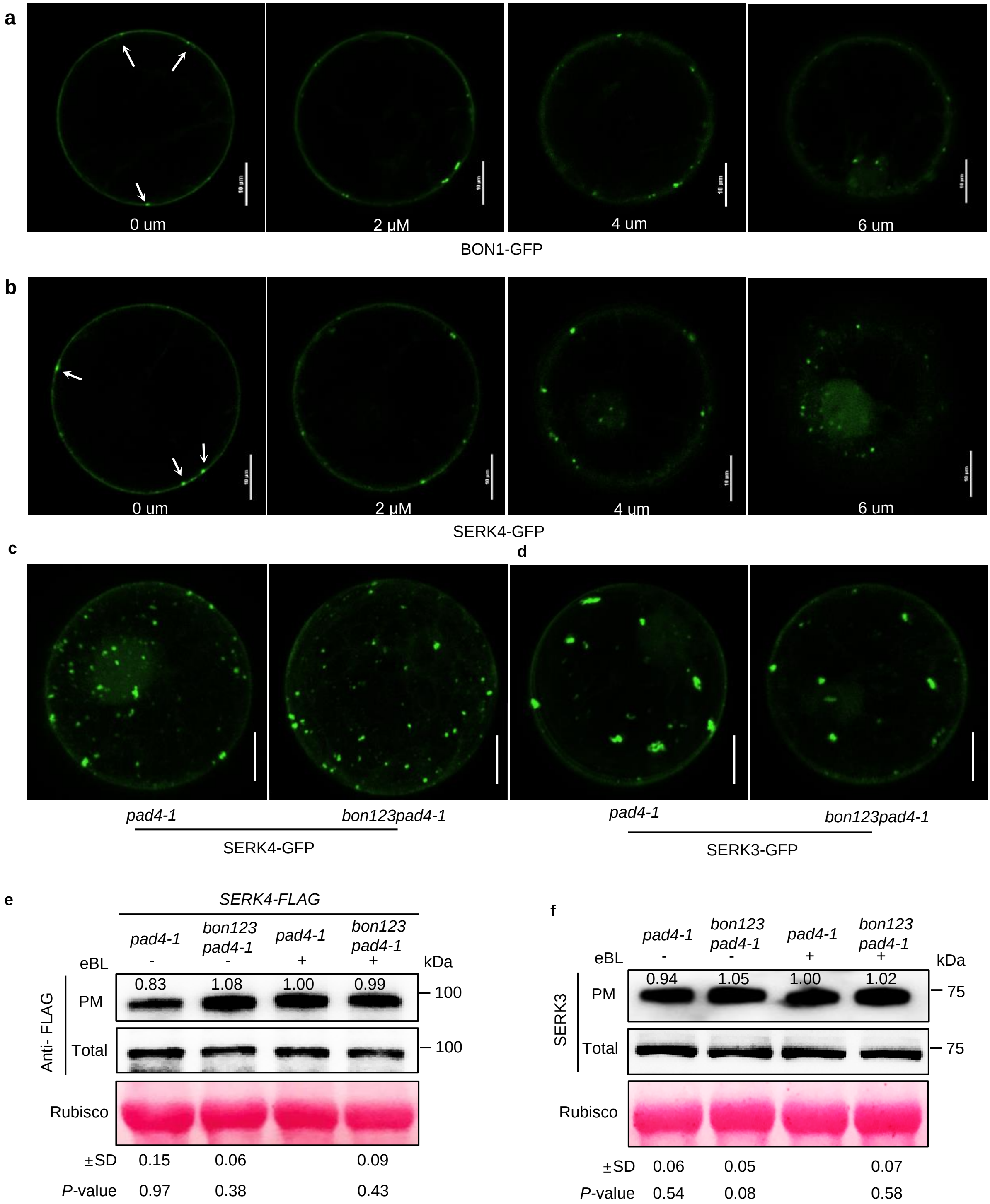

Supplementary Fig. 15|Protein trafficking of SERK4 and SERK3 proteins in *pad4-1* and *bon1-1 bon2-2 bon3-3 pad4-1*. a, **b** The subcellular localization of BON1 (**a**) or SERK4 (**b**) in Arabidopsis protoplasts. BON1 (**a**) or SERK4 (**b**) was fused with GFP and transiently expressed in Arabidopsis protoplasts. The white arrowheads point to the vesicles budding from the plasma membrane. A set of the cross-sectional images are shown at the indicated depth (0 μm, 2 μm, 4 μm and 6 μm). **c**, **d** Subcellular localization of SERK4 (**c**) or SERK3 (**d**) in the protoplasts of *pad4-1* and *bon1-1 bon2-2 bon3-3 pad4-1* (*bon123pad4-1*) after eBL treatment. The images were 3D-projection graphs stacked with multiple focal plane layers. Scale bars, 10 μm. **e**, **f** Protein abundance of SERK4 (**e**) or SERK3 (**f**) in *pad4-1* and *bon1-1 bon2-2 bon3-3 pad4-1* (*bon123pad4-1*) seedlings before (-) and after (+) BL treatment detected by immunoblot. 'PM' represents isolated plasma membrane proteins. 'Total' represents total proteins of seedlings. The Ponceau S staining (Rubisco) shows equal protein loading. Numbers underlying each band denote the relative intensity of PM protein normalized with the corresponding band of total protein by Image J quantification. Intensity of SERK4 (**e**) or SERK3 (**f**) in PM in *pad4-1* after BL treatment was set to 1.00. Data are shown as mean ± SD from three replicates. Significant differences were determined by two-tailed Student's *t*-tests at $P < 0.05$ (n = 3, ±SD) in **e** and **f**.

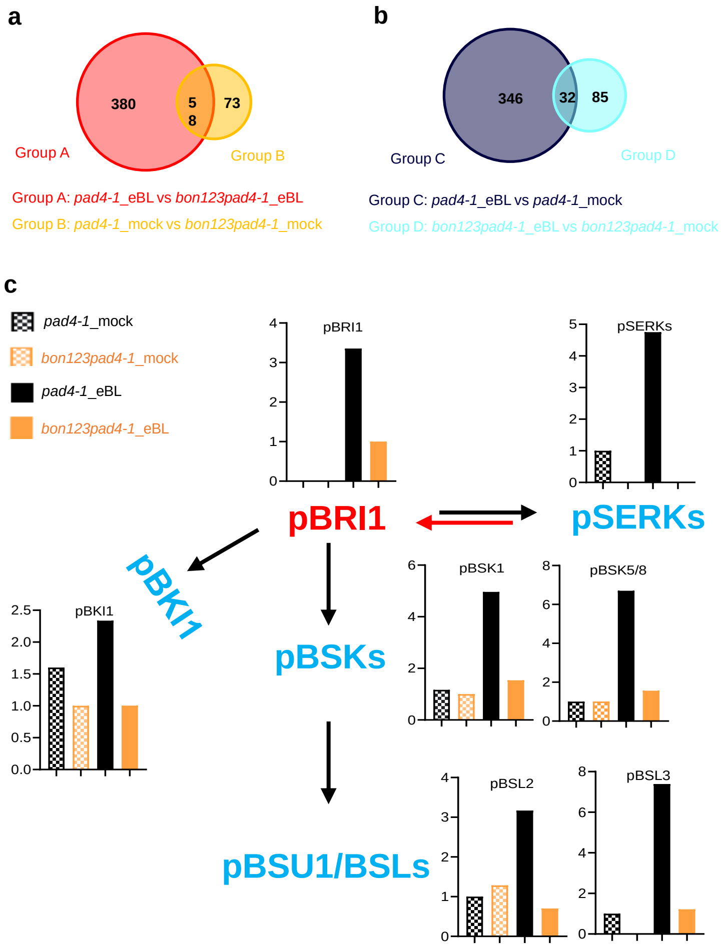

Supplementary Fig. 16|Analysis of differentially phosphorylated proteins in *pad4-1*_eBL vs *bon1-1 bon2-2 bon3-3 pad4-1*_eBL. a, **b** Venn diagram showing the number of differentially phosphorylated proteins in *pad4-1*_eBL vs *bon1-1 bon2-2 bon3-3 pad4-1*_eBL (Group A) and *pad4-1*_mock vs *bon1-1 bon2-2 bon3-3 pad4-1*_mock (Group B) (**a**), or in *pad4-1*_eBL vs *pad4-1*_mock (Group C) and *bon1-1 bon2-2 bon3-3 pad4-1*_eBL vs *bon1-1 bon2-2 bon3-3 pad4-1*_mock (Group D) (**b**). **c** The relative intensity of phosphorylated peptides of BRI1, SERK1/2/3, BKI1, BSK1, BSK5/8, BSL2 and BSL3.

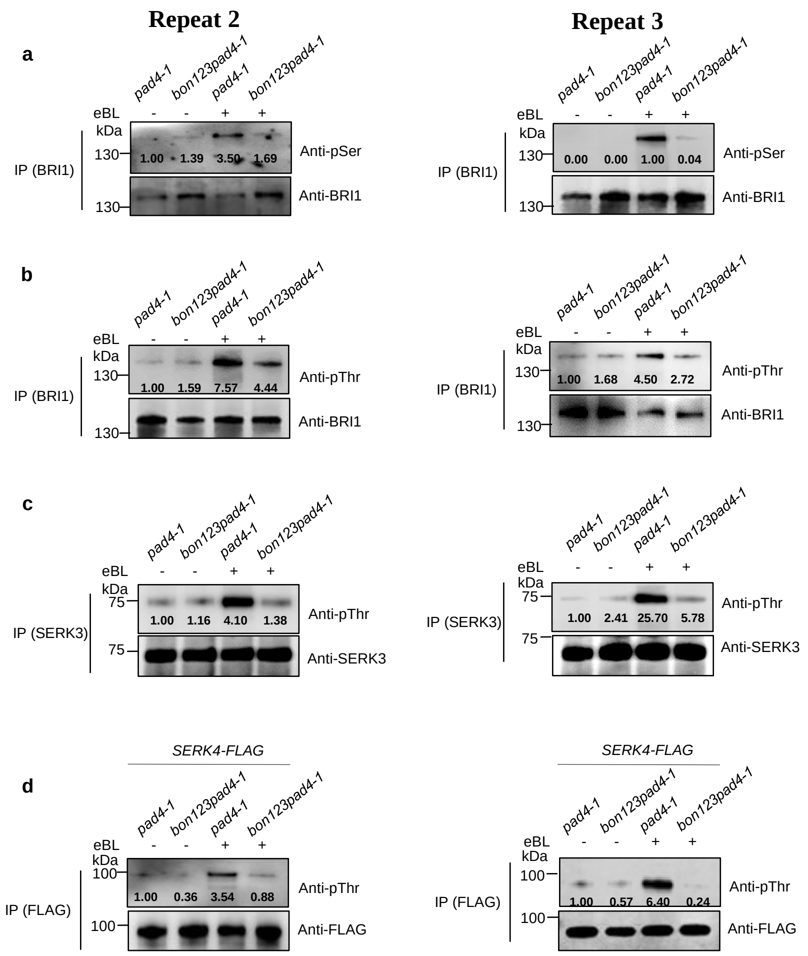

Supplementary Fig. 17|Two additional biological replicates to detect the phosphorylation levels of BRI1 and SERKs in *pad4-1* and *bon1-1 bon2-2 bon3-3 pad4-1* mutant plants by immunoblots. **a**, **b**, **c**, **d** Phosphorylation levels of BRI1 (**a**, **b**), SERK3 (**c**) and SERK4 (**d**) in the *pad4-1* and *bon1-1 bon2-2 bon3-3 pad4-1* (*bon123pad4-1*) mutants without or with eBL treatment. Phosphorylated proteins were detected with anti-pSer and anti-pThr antibodies. Numbers underlying each band denote their relative intensity normalized with the corresponding band of total immunoprecipitated proteins by ImageJ quantification.

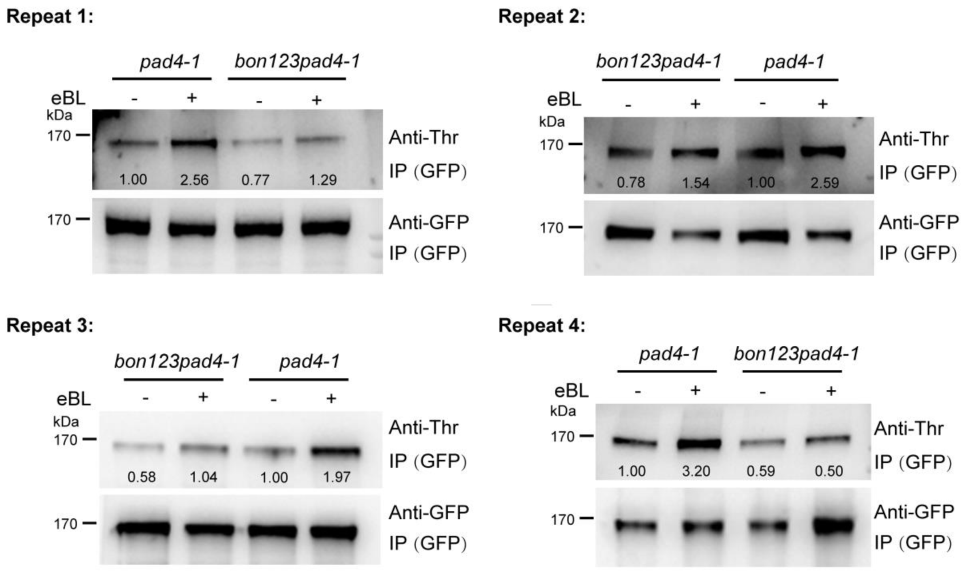

Supplementary Fig. 18|Phosphorylation levels of BRI1-GFP in *pad4-1* and *bon1-1 bon2-2 bon3-3 pad4-1* protoplasts without or with eBL treatment. Phosphorylated proteins were detected with anti-pThr antibody. Four independent experimental repeats are shown with similar trends. Numbers underlying each band denote their relative intensity normalized with the corresponding band of total immunoprecipitated proteins by ImageJ quantification.

Supplementary Table 1. Primers used in this study

Name	Sequence 5'-3'	Characteristics
ZmBON1_MT1T2-BsF	AATAATGGTCTCAGGCGATCGCTTTCTCCGCTCCCG	for pBUE411-2gR-ZmBON1
ZmBON1_MT1T2-F0	GATCGCTTTCTCCGCTCCCGGTTTTAGAGCTAGAAATAGC	
ZmBON1_MT1T2-R0	GAACTTTCTTCAGCCTGAACGCTTCTTGGTGCC	
ZmBON1_MT1T2-BsR	ATTATTGGTCTCTAAACGAACTTTCTTCAGCCTGAA	
ZmBON3_MT1T2-BsF	AATAATGGTCTCAGGCGTGGTCTACACTAACATCAA	for pBUE411-2gR-ZmBON3
ZmBON3_MT1T2-F0	GTGGTCTACACTAACATCAAGTTTTAGAGCTAGAAATAGC	
ZmBON3_MT1T2-R0	CGCGTCTGGAAGAAATAGTCGCTTCTTGGTGCC	
ZmBON3_MT1T2-BsR	ATTATTGGTCTCTAAACCGCGTCTGGAAGAAATAGT	
ZmBON1_MT1T2-BsF	AATAATGGTCTCAGGCGATCGCTTTCTCCGCTCCCG	for pBUE411-2gR-ZmBON1/3
ZmBON1_MT1T2-F0	GATCGCTTTCTCCGCTCCCGGTTTTAGAGCTAGAAATAGC	
ZmBON3_MT1T2-R0	CGCGTCTGGAAGAAATAGTCGCTTCTTGGTGCC	
ZmBON3_MT1T2-BsR	ATTATTGGTCTCTAAACCGCGTCTGGAAGAAATAGT	
BAR3-F	CCATCGTCAACCACTACATCGAGACA	genotyping transgenic line, fwd
BAR3-R	CTTCAGCAGGTGGGTGTAGAGCGT	genotyping transgenic line, rev
ZmBON1_T1-F	GGGTCTCCGTGTCGTCTCTGCGA	genotyping *zmbon1* target1,fwd
ZmBON1_T1-R	GCCAACGAGTAACGTAGCAGTA	genotyping *zmbon1* target1,rev
ZmBON1_T2-F	GTAGCTTGTTCTCTAGTGGCCTA	genotyping *zmbon1* target2,fwd
ZmBON1_T2-R	GTAATGCGCCAGCTGTTGATGGT	genotyping *zmbon1* target2,rev
ZmBON3_T1-F	TCCGTGTACGACGGTCAGGCTCA	genotyping *zmbon3* target1,fwd
ZmBON3_T1-R	GCAGTTTTGGTGATCAGGCCGGA	genotyping *zmbon3* target1,rev
ZmBON3-T2-F	TATTGCTATGCCTGTATTTTACC	genotyping *zmbon3* target2,fwd
ZmBON3-T2-R	TTCCACAAACAGAACTTGAGAGA	genotyping *zmbon3* target2,rev
EMS3-05dba0-F	TTCAAGTATTAATGGCCCAGGCT	genotyping $zmbon1^{EMS}$,fwd
EMS3-05dba0-R	TGGTACGTATTTGCGATTGCTCT	genotyping $zmbon1^{EMS}$,rev
EMS3-06b988-F	TAAACACCACCATTAAGAGAATA	genotyping $zmbon3^{EMS}$,fwd
EMS3-06b988-R	TAACTGATGACAGATGAAAGAAC	genotyping $zmbon3^{EMS}$,rev
GRMZM2G082207-F	GTCAACAGAAGTTGACGTGAGCATT	off-target detection,fwd
GRMZM2G082207-R	AAAGCACACGCCACCGCACGAAAAC	off-target detection,rev
GRMZM5G852170-F	CTATTGGGGAGGGTCCTCTTGCCAC	off-target detection,fwd
GRMZM5G852170-R	CTGGCTCCTCGTCCGAAAACACCCT	off-target detection,rev
GRMZM2G064278-F	CCGCCGCTGATTTCTCGGCGGCGGC	off-target detection,fwd
GRMZM2G064278-R	GGGGCTGCTGTTCCACTCGTTCTGC	off-target detection,rev
qZmPR1-F	CGCGAGAGCCCCTACTAGAC	ZmPR1 qPCR, fwd
qZmPR1-R	AAATCGCCTGCATGGTTTTA	ZmPR1 qPCR, rev
qZmPR5-F	GTCATCGACGGCTACAACCT	ZmPR5 qPCR, fwd
qZmPR5-R	CACGGGCAGAAGGTGACT	ZmPR5 qPCR, rev
qZmACTIN1-F	ATCCAGGCTGTTCTTTCGTT	ZmACTIN1 qPCR, fwd
qZmACTIN1-R	CATTAGGTGGTCGGTGAGGT	ZmACTIN1 qPCR, rev
qZmUBQ-F	TAAGCTGCCGATGTGCCTGCGTCG	ZmUBQ qPCR, fwd
qZmUBQ-R	CTGAAAGACAGAACATAATGAGCACAG	ZmUBQ qPCR, rev
qZmCPD-F	GATAGAGTAAGGTGGATGTCGTG	ZmCPD qPCR, fwd
qZmCPD-R	GTCCAGGTTACAGAGCAAGAG	ZmCPD qPCR, rev
qZmBRD1-F	GAAATAACCCATGCGATGCTG	ZmBRD1 qPCR, fwd
qZmBRD1-R	ACATTACTATCGGCATGGACAC	ZmBRD1 qPCR, rev

qACTIN-F	GACCAGCTCTTCCATCGAGAA	ACTIN qPCR, fwd
qACTIN-R	CAAACGAGGGCTGGAACAAG	ACTIN qPCR, rev
qCPD-F	CCTTTCATCGACGAGAGAGTAG	CPD qPCR, fwd
qCPD-R	GAGTGTTTCCCCAAAAGGTTAC	CPD qPCR, rev
qROT3-F	TTTGTCCCTGCATACCCTAAAT	ROT3 qPCR, fwd
qROT3-R	TCTCATCCTGAACATGAACCAA	ROT3 qPCR, rev
qBR6OX2-F	GGGAGTTTCTTCAAGTCTCACA	BR6OX2 qPCR, fwd
qBR6OX2-R	GCAACAAGTCCTTTCGATTCAT	BR6OX2 qPCR, rev
qDWF4-F	CATCAGTTAAAAGCATCTCCGG	DWF4 qPCR, fwd
qDWF4-R	CTCAAAGCCTTGTTGATTCACA	DWF4 qPCR, rev
qBAS1-F	AGGACCATGTCGTTAAGCTTAA	BAS1 qPCR, fwd
qBAS1-R	GACCGCTATGATTGGGATTAGA	BAS1 qPCR, rev
qSAUR-AC1-F	TGGGTGCTAAGCAAATTATTCG	SAUR-AC1 qPCR, fwd
qSAUR-AC1-R	TGAGATGTGACTGTGAAGAACA	SAUR-AC1 qPCR, rev
pGBKT7-AtBON1A-F	atggccatggaggccgaattcATGGTGGACAAGTTTACCGAGACT	BD-AtBON1-A fusion in pGBKT7,fwd
pGBKT7-AtBON1A-R	ccgctgcaggtcgacggatccCAATAGAAGAAATTAAGCTGCACAAA	BD-AtBON1A fusion in pGBKT7, rev
pGADT7-AtBKK1-F	gccatggaggccagtgaattcATGACAAGTTCAAAAATGGAACAAAG	AD-AtBKK1-KD fusion in pGADT7,fwd
pGADT7-AtBKK1-R	cagctcgagctcgatggatccTTATCTTGGACCCGAGGGGTAA	AD-AtBKK1-KD fusion in pGADT7, rev
pGADT7-AtSERK1_KD-F	gccatggaggccagtgaattcATGCGACGAAGAAAGCCACTAGATAT	AD-AtSERK1-KDfusion in pGADT7,fwd
pGADT7-AtSERK1_KD-R	cagctcgagctcgatggatccTTACCTTGGACCAGATAACTCAACG	AD-AtSERK1-KD fusion in pGADT7, rev
pGADT7-AtSERK2_KD-F	gccatggaggccagtgaattcATGCGTAGAAGAAAACCTCAAGAATTC	AD-AtSERK2-KD fusion in pGADT7,fwd
pGADT7-AtSERK2_KD-R	cagctcgagctcgatggatccTTATCTTGGACCAGACAACTCC	AD-AtSERK2-KD fusion in pGADT7, rev
pGADT7-AtBRI1KD-F	gccatggaggccagtgaattcATGGGTTTCCATAATGATAGTCTGATT	AD-AtBRI1-KD fusion in pGADT7,fwd
pGADT7-AtBRI1KD-R	cagctcgagctcgatggatccTCATAATTTTCCTTCAGGAACTTCTTT	AD-AtBRI1-KD fusion in pGADT7, rev
pGADT7-AtBON1A-F	gccatggaggccagtgaattcATGGTGGACAAGTTTACCGAGACT	AD-AtBON1-A fusion in pGADT7,fwd
pGADT7-AtBON1A-R	cagctcgagctcgatggatccCAATAGAAGAAATTAAGCTGCACAAA	AD-AtBON1A fusion in pGADT7, rev
pGBKT7-AtTTL1-F	atggccatggaggccgaattcATGCCCAAGTCAGTTAAACCCAT	AD-AtTTL1 fusion in pGADT7,fwd
pGBKT7-AtTTL1-R	ccgctgcaggtcgacggatccTTAACCGCTATAGTGTCTCACCG	AD-AtTTL1 fusion in pGADT7, rev
pGBKT7-AtTTL4-F	atggccatggaggccgaattcATGTCACATTATAGAAGACATTCGC	AD-AtTTL4 fusion in pGADT7,fwd
pGBKT7-AtTTL4-R	ccgctgcaggtcgacggatccTTATAAGAGGAAATGCGTAACAGAGT	AD-AtTTL4 fusion in pGADT7, rev
pGBKT7-AtBKI1-F	atggccatggaggccgaattcATGGAAACTAATCTACAACAGGTGAAG	AD-AtBKI1 fusion in pGADT7,fwd
pGBKT7-AtBKI1-R	ccgctgcaggtcgacggatccTCAAGAATCCTTAACCTTATCATCACG	AD-AtBKI1 fusion in pGADT7, rev
pGBKT7-AtBIK1-F	atggccatggaggccgaattcATGGGTTCTTGCTTCAGTTCTCG	AD-AtBIK1 fusion in pGADT7,fwd
pGBKT7-AtBIK1-R	ccgctgcaggtcgacggatccCTACACAAGGTGCCTGCCAA	AD-AtBIK1 fusion in pGADT7, rev
pGBKT7-AtBSK1-F	atggccatggaggccgaattcATGGGTTGTTGTCAATCCTTGTT	AD-AtBSK1 fusion in pGADT7,fwd
pGBKT7-AtBSK1-R	ccgctgcaggtcgacggatccTCAAGATCCTCTGCCGCCT	AD-AtBSK1 fusion in pGADT7, rev
pGBKT7-AtBSK3-F	atggccatggaggccgaattcATGGGAGGTCAATGCTCTAG	AD-AtBSK3 fusion in pGADT7,fwd
pGBKT7-AtBSK3-R	ccgctgcaggtcgacggatccTTACTTCACTCGGGGAACTC	AD-AtBSK3 fusion in pGADT7, rev
pGBKT7-AtBIN2-F	atggccatggaggccgaattcATGGCTGATGATAAGGAGATGCC	AD-AtBIN2 fusion in pGADT7,fwd
pGBKT7-AtBIN2-R	ccgctgcaggtcgacggatccTTAAGTTCCAGATTGATTCAAGAAGC	AD-AtBIN2 fusion in pGADT7, rev
pGBKT7_ZmBON1A-F	atggccatggaggccgaattcATGGTGTTGAAGAGCCAGCTATATGTA GAG	AD fusion in pGADT7,fwd
pGBKT7_ZmBON1A-R	ccgctgcaggtcgacggatccCATGAATTGCCCTGGTACCTCA	AD fusion in pGADT7, rev
pGADT7_ZmBKK1KD-F	gccatggaggccagtgaattcATGTTCAGCAATAAGAACATTCTTGGAA	AD fusion in pGADT7,fwd
pGADT7_ZmBKK1KD-R	cagctcgagctcgatggatccTTGAACAGAGGAAATGGCACAG	AD fusion in pGADT7, rev
AtBON1-cLuc-F	gcgtcccggggcggtaccATGGGGAATTGTTGCTCCGATG	cLuc fusion with AtBON1,fwd
AtBON1-cLuc-R	cgaaagctctgcaggtcgacTGGAGGAATCGGTTTCATA	cLuc fusion with AtBON1,rev

AtBKK1-nLuc-F	ggggacgagctcggtaccATGACAAGTTCAAAAATGGAACAAAG	nLuc fusion with AtBKK1,fwd
AtBKK1-nLuc-R	gtacgagatctggtcgacaTCTTGGACCCGAGGGGTAA	nLuc fusion with AtBKK1,rev
AtBRI1-nLuc-F	ggggacgagctcggtaccATGAAGACTTTTTCAAGCTTCTT	nLuc fusion with AtBRI1,fwd
AtBR11-nLuc-R	gtacgagatctggtcgacaTAATTTTCCTTCAGGAACTTCTT	nLuc fusion with AtBRI1,rev
ZmBON1-cLuc-F	gcgtcccggggcggtaccATGGGGAACTGCTGCTCCGAT	cLuc fusion with ZmBON1,fwd
ZmBON1-cLuc-R	cgaaagctctgcaggtcgacACTGTCTGTTTGGGTTTCTC	cLuc fusion with ZmBON1,rev
ZmBKK1-nLuc-F	ggggacgagctcggtaccATGGCTGCGGCGGAGGCG	nLuc fusion with ZmBKK1,fwd
ZmBKK1-nLuc-R	gtacgagatctggtcgacaACAATTGCATAGGAAAAGGTGAGCA	nLuc fusion with ZmBKK1,rev
psuper-1300-AtBKK1-F	atacaccaaatcgactctagaATGACAAGTTCAAAAATGGAACAAAG	FLAG fusion with AtBKK1,fwd
psuper-1300-AtBKK1-STOP-R	gtctttgtaatccatggtaccTCTTGGACCCGAGGGGTAA	FLAG fusion with AtBKK1,rev
p2300-GFP-AtBRI1-F	atttggagaggacagggtaccATGAAGACTTTTTCAAGCTTCTT	GFP fusion with AtBRI1,fwd
p2300-GFP-AtBRI1-STOP-R	cttgctcaccatggtactagtTAATTTTCCTTCAGGAACTTCTT	GFP fusion with AtBRI1,rev

Supplementary Table 2. Maxquant identification and quantitation indexes

Item	Value
Enzyme	Trypsin
Max Missed Cleavages	2
Main search （mass tolerance of precursor ions)	6 ppm
First search （mass tolerance of precursor ions)	20 ppm
MS/MS Tolerance （mass tolerance of MS2)	20 ppm
Fixed modifications	Carbamidomethyl (C)
Variable modifications	Oxidation (M), Phospho (STY)
Database pattern	Reverse
Include contaminants	TRUE
Peptide FDR	⩽0.01
Site FDR	⩽0.01
Protein FDR	⩽0.01
Time window (match between runs)	2min
