# Supplementary material for: Copine proteins are required for brassinosteroid signaling in maize and Arabidopsis
Source: Nat Commun. 2024 Mar 8;15:2028. doi: 10.1038/s41467-024-46289-6 (PMC10923931; doi:10.1038/s41467-024-46289-6)
Supplement: Supplementary file 3 — Description of Additional Supplementary Files [file 41467_2024_46289_MOESM3_ESM.pdf]

Description of Additional Supplementary Files

Supplementary Data 1 : Differentially expressed genes in the wild-type KN5585 vs *Zmbon1*.

Supplementary Data 2 : Transcriptomics data of all expressed genes in Col-0, *bak1-4*, *pad4-1* and *bon1-1 bon2-2 bon3-3 pad4-1* under mock or eBL treatment.

Supplementary Data 3 : Differentially expressed genes in *pad4-1*, *bon1-1 bon2-2 bon3-3 pad4-1*, Col-0 and *bak1-4* under mock and eBL treatment.

Supplementary Data 4 : Differentially phosphorylated peptides in *pad4-1* vs *bon1-1 bon2-2 bon3-3 pad4-1* under mock and eBL treatment.

Supplementary Data 5 : All phosphorylated peptides detected in *pad4-1* and *bon1-1 bon2-2 bon3-3 pad4-1* under mock or eBL treatment.
