# Supplementary material for: Copine proteins are required for brassinosteroid signaling in maize and Arabidopsis
Source: Nat Commun. 2024 Mar 8;15:2028. doi: 10.1038/s41467-024-46289-6 (PMC10923931; doi:10.1038/s41467-024-46289-6)
Supplement: Supplementary file 9 — Reporting Summary [file 41467_2024_46289_MOESM9_ESM.pdf]

nature portfolio

Corresponding author(s): Mingyue Gou

Last updated by author(s): Feb 13, 2024

Reporting Summary

Nature Portfolio wishes to improve the reproducibility of the work that we publish. This form provides structure for consistency and transparency in reporting. For further information on Nature Portfolio policies, see our Editorial Policies and the Editorial Policy Checklist.

Statistics

For all statistical analyses, confirm that the following items are present in the figure legend, table legend, main text, or Methods section.

n/a	Confirmed	
☐	☒	The exact sample size (*n*) for each experimental group/condition, given as a discrete number and unit of measurement
☐	☒	A statement on whether measurements were taken from distinct samples or whether the same sample was measured repeatedly
☐	☒	The statistical test(s) used AND whether they are one- or two-sided<br>*Only common tests should be described solely by name; describe more complex techniques in the Methods section.*
☒	☐	A description of all covariates tested
☒	☐	A description of any assumptions or corrections, such as tests of normality and adjustment for multiple comparisons
☐	☒	A full description of the statistical parameters including central tendency (e.g. means) or other basic estimates (e.g. regression coefficient) AND variation (e.g. standard deviation) or associated estimates of uncertainty (e.g. confidence intervals)
☐	☒	For null hypothesis testing, the test statistic (e.g. *F*, *t*, *r*) with confidence intervals, effect sizes, degrees of freedom and *P* value noted<br>*Give P values as exact values whenever suitable.*
☒	☐	For Bayesian analysis, information on the choice of priors and Markov chain Monte Carlo settings
☒	☐	For hierarchical and complex designs, identification of the appropriate level for tests and full reporting of outcomes
☒	☐	Estimates of effect sizes (e.g. Cohen's *d*, Pearson's *r*), indicating how they were calculated

Our web collection on statistics for biologists contains articles on many of the points above.

Software and code

Policy information about availability of computer code

Data collection	Total SA were detected by MetWare (http://www.metware.cn/) based on the AB Sciex QTRAP 6500 LC-MS/MS platform;Total RNA were used for sequencing on an Illumina NovaSeq 6000 platform; Mass spectrometry analysis using a timsTOF Pro mass spectrometer (Bruker) coupled to a Nanoelute (Bruker Daltonics);The luciferase activity was imaged with a NightShade LB985 Plant Imaging System (Berthold Technologies);Real-time expression data were examined using the Roche LightCycler® 96 thermal cycler instrument (Roche Applied Science); Localization of all fusion proteins were examined under confocal microscopy (Nikon A1+);
Data analysis	Phylogenetic analysis of BON1 homologs was analyzed using the MEGA7.0.26 software; Alignment of the full-length amino acid sequences of BONZAI by DNAMAN (Vesion 9); Heatmap and Venn diagrams were performed by using TBtools-II;Statistical enrichment of DEGs in Kyoto Encyclopedia of Genes and Genomes (KEGG) pathways was performed using KOBAS software;The phosphoproteomics data were analyzed and quantified using MaxQuant software;Protein immunoblotting band intensity was determined by ImageJ2.1.0; All box plot and column diagram were analyzed by drawn GraphPad Prism software8.

For manuscripts utilizing custom algorithms or software that are central to the research but not yet described in published literature, software must be made available to editors and reviewers. We strongly encourage code deposition in a community repository (e.g. GitHub). See the Nature Portfolio guidelines for submitting code & software for further information.

Data

Policy information about availability of data

All manuscripts must include a data availability statement. This statement should provide the following information, where applicable:

- Accession codes, unique identifiers, or web links for publicly available datasets
- A description of any restrictions on data availability
- For clinical datasets or third party data, please ensure that the statement adheres to our policy

The transcriptomics data for maize and Arabidopsis of this article are available in the NCBI database under the accession numbers PRJNA907414 (https://www.ncbi.nlm.nih.gov/bioproject/PRJNA907414/) and PRJNA907415 (https://www.ncbi.nlm.nih.gov/bioproject/?term=PRJNA907415). The phosphoproteomics data of this article are available in the iProX database under the accession number IPX0005474000 (https://www.iprox.cn/page/project.html?id=IPX0005474000). The whole transcriptomic data for Col-0, bak1-4, pad4-1 and bon1bon2bon3pad4-1 and the whole phosphoproteomic data for pad4-1 and bon1bon2bon3pad4-1 under mock and eBL treatment are provided as Supplementary Data Files. Source data are provided with this paper

Research involving human participants, their data, or biological material

Policy information about studies with human participants or human data. See also policy information about sex, gender (identity/presentation), and sexual orientation and race, ethnicity and racism.

Reporting on sex and gender	NA
Reporting on race, ethnicity, or other socially relevant groupings	NA
Population characteristics	NA
Recruitment	NA
Ethics oversight	NA

Note that full information on the approval of the study protocol must also be provided in the manuscript.

Field-specific reporting

Please select the one below that is the best fit for your research. If you are not sure, read the appropriate sections before making your selection.

☒ Life sciences ☐ Behavioural & social sciences ☐ Ecological, evolutionary & environmental sciences

For a reference copy of the document with all sections, see nature.com/documents/nr-reporting-summary-flat.pdf

Life sciences study design

All studies must disclose on these points even when the disclosure is negative.

Sample size	To exclude random effects, independent replicates were performed in this study.
Data exclusions	No data were excluded from the analysis.
Replication	The number of replicates (n) in Figure 1b, Figure 2d, Figure 3c and 3e, and Extended Data Figure 8b, Extended Data Figure 9c were directly displayed in the Figure;There are at least three biological replicates in Figure 1e and 1f, Figure 2b and 2e, Figure 4e, Figure 6a-d, Figure 6f-i and Extended data Figure 18;<br>For RNA-seq, we used three biological replicates.
Randomization	For phenotypic observations, maize (inbred lines KN5585 and B73) plants were planted in the field in Zhengzhou in summer or Sanya in winter from 2019 to 2022. For laboratory experiments, maize plants were grown at 22? under long-day conditions (14-h day/10-h night) in a growth chamber. Arabidopsis seedlings from the Col-0 and Ws accessions used in this study were grown at 22? under long-day conditions (14-h day/10-h night) in chambers. Plants were randomly used for experiments.
Blinding	All data so blinding is not possible, but we try to be objective by multiple indepentent experiments in this study.

Reporting for specific materials, systems and methods

We require information from authors about some types of materials, experimental systems and methods used in many studies. Here, indicate whether each material, system or method listed is relevant to your study. If you are not sure if a list item applies to your research, read the appropriate section before selecting a response.

Materials & experimental systems

n/a	Involved in the study
☐	☒ Antibodies
☐	☐ Eukaryotic cell lines
☐	☐ Palaeontology and archaeology
☐	☐ Animals and other organisms
☐	☐ Clinical data
☐	☐ Dual use research of concern
☐	☐ Plants

Methods

n/a	Involved in the study
☐	☐ ChIP-seq
☐	☐ Flow cytometry
☐	☐ MRI-based neuroimaging

Antibodies

Antibodies used: anti-BZR1 (Youke Biotechnology, Shanghai, China, 454 YKRP082); anti-BRI1 (Agrisera, AS12 1859); anti-BAK1 (Agrisera, 455 AS12 1858); anti-FLAG (Sigma, F1804); anti-phosphoserine (Sigma, 456 P5747); anti-phosphothreonine (Cell signaling, 9381); anti-HA (Covance, 457 MMS-101R); anti-GFP (TransGen Biotech, HT801); anti-rabbit (MBL, 458); and anti-mouse (Solarbio, 458 SE131).

Validation: The validation of primary antibody were provided in the Manuscript (Supplementary Fig.13).

Eukaryotic cell lines

Policy information about cell lines and Sex and Gender in Research

Cell line source(s): NA

Authentication: NA

Mycoplasma contamination: NA

Commonly misidentified lines (See ICLAC register): NA

Palaeontology and Archaeology

Specimen provenance: NA

Specimen deposition: NA

Dating methods: NA

☐ Tick this box to confirm that the raw and calibrated dates are available in the paper or in Supplementary Information.

Ethics oversight: NA

Note that full information on the approval of the study protocol must also be provided in the manuscript.

Animals and other research organisms

Policy information about studies involving animals; ARRIVE guidelines recommended for reporting animal research, and Sex and Gender in Research

Laboratory animals: NA

Wild animals: NA

Reporting on sex: NA

Field-collected samples: NA

Ethics oversight: NA

Note that full information on the approval of the study protocol must also be provided in the manuscript.

Clinical data

Policy information about clinical studies

All manuscripts should comply with the ICMJE guidelines for publication of clinical research and a completed CONSORT checklist must be included with all submissions.

Clinical trial registration	NA
Study protocol	NA
Data collection	NA
Outcomes	NA

Dual use research of concern

Policy information about dual use research of concern

Hazards

Could the accidental, deliberate or reckless misuse of agents or technologies generated in the work, or the application of information presented in the manuscript, pose a threat to:

No	Yes	
☒	☐	Public health
☒	☐	National security
☒	☐	Crops and/or livestock
☒	☐	Ecosystems
☒	☐	Any other significant area

Experiments of concern

Does the work involve any of these experiments of concern:

No	Yes	
☒	☐	Demonstrate how to render a vaccine ineffective
☒	☐	Confer resistance to therapeutically useful antibiotics or antiviral agents
☒	☐	Enhance the virulence of a pathogen or render a nonpathogen virulent
☒	☐	Increase transmissibility of a pathogen
☒	☐	Alter the host range of a pathogen
☒	☐	Enable evasion of diagnostic/detection modalities
☒	☐	Enable the weaponization of a biological agent or toxin
☒	☐	Any other potentially harmful combination of experiments and agents

Plants

Seed stocks	Zmbon1-7 (EMS3-05dba0) and Zmbon3-7 (EMS3-06b988) EMS mutants were purchased from MEMD (http://www.elabcaas.cn/memd/public/index.html#/).
Novel plant genotypes	To generate knock-out mutants of Zmbon1, Zmbon3 and Zmbon1/3, two single guide RNA sequences (sgRNAs) each targeting ZmBON1 or ZmBON3 were cloned into the pBUE411 vector to generate the CRISPR/Cas9 constructs targeting ZmBON1 and ZmBON3 . PCR products covering the target site in the respective genes were sequenced to determine the genotype of all plants. The detail information about the targeting guide RNA sequence for ZmBON1 and ZmBON3 are showed in Supplementary Fig.2.
Authentication	Off-target detection of Zmbon1. No mutations were found in the three predicted off-target genes GRMZM2G082207, GRMZM5G852170, and GRMZM2G064278.

ChIP-seq

Data deposition

- [ ] Confirm that both raw and final processed data have been deposited in a public database such as GEO.
- [ ] Confirm that you have deposited or provided access to graph files (e.g. BED files) for the called peaks.

Data access links<br>*May remain private before publication.*	*For "Initial submission" or "Revised version" documents, provide reviewer access links. For your "Final submission" document, provide a link to the deposited data.*
Files in database submission	*Provide a list of all files available in the database submission.*
Genome browser session<br>(e.g. UCSC)	*Provide a link to an anonymized genome browser session for "Initial submission" and "Revised version" documents only, to enable peer review. Write "no longer applicable" for "Final submission" documents.*

Methodology

Replicates	*Describe the experimental replicates, specifying number, type and replicate agreement.*
Sequencing depth	*Describe the sequencing depth for each experiment, providing the total number of reads, uniquely mapped reads, length of reads and whether they were paired- or single-end.*
Antibodies	*Describe the antibodies used for the ChIP-seq experiments; as applicable, provide supplier name, catalog number, clone name, and lot number.*
Peak calling parameters	*Specify the command line program and parameters used for read mapping and peak calling, including the ChIP, control and index files used.*
Data quality	*Describe the methods used to ensure data quality in full detail, including how many peaks are at FDR 5% and above 5-fold enrichment.*
Software	*Describe the software used to collect and analyze the ChIP-seq data. For custom code that has been deposited into a community repository, provide accession details.*

Flow Cytometry

Plots

Confirm that:

- [ ] The axis labels state the marker and fluorochrome used (e.g. CD4-FITC).
- [ ] The axis scales are clearly visible. Include numbers along axes only for bottom left plot of group (a 'group' is an analysis of identical markers).
- [ ] All plots are contour plots with outliers or pseudocolor plots.
- [ ] A numerical value for number of cells or percentage (with statistics) is provided.

Methodology

Sample preparation	*Describe the sample preparation, detailing the biological source of the cells and any tissue processing steps used.*
Instrument	*Identify the instrument used for data collection, specifying make and model number.*
Software	*Describe the software used to collect and analyze the flow cytometry data. For custom code that has been deposited into a community repository, provide accession details.*
Cell population abundance	*Describe the abundance of the relevant cell populations within post-sort fractions, providing details on the purity of the samples and how it was determined.*
Gating strategy	*Describe the gating strategy used for all relevant experiments, specifying the preliminary FSC/SSC gates of the starting cell population, indicating where boundaries between "positive" and "negative" staining cell populations are defined.*

- [ ] Tick this box to confirm that a figure exemplifying the gating strategy is provided in the Supplementary Information.

Magnetic resonance imaging

Experimental design

Design type	*Indicate task or resting state; event-related or block design.*

Design specifications | *Specify the number of blocks, trials or experimental units per session and/or subject, and specify the length of each trial or block (if trials are blocked) and interval between trials.*

Behavioral performance measures | *State number and/or type of variables recorded (e.g. correct button press, response time) and what statistics were used to establish that the subjects were performing the task as expected (e.g. mean, range, and/or standard deviation across subjects).*

Acquisition

Imaging type(s) | *Specify: functional, structural, diffusion, perfusion.*

Field strength | *Specify in Tesla*

Sequence & imaging parameters | *Specify the pulse sequence type (gradient echo, spin echo, etc.), imaging type (EPI, spiral, etc.), field of view, matrix size, slice thickness, orientation and TE/TR/flip angle.*

Area of acquisition | *State whether a whole brain scan was used OR define the area of acquisition, describing how the region was determined.*

Diffusion MRI ☐ Used ☐ Not used

Preprocessing

Preprocessing software | *Provide detail on software version and revision number and on specific parameters (model/functions, brain extraction, segmentation, smoothing kernel size, etc.).*

Normalization | *If data were normalized/standardized, describe the approach(es): specify linear or non-linear and define image types used for transformation OR indicate that data were not normalized and explain rationale for lack of normalization.*

Normalization template | *Describe the template used for normalization/transformation, specifying subject space or group standardized space (e.g. original Talairach, MNI305, ICBM152) OR indicate that the data were not normalized.*

Noise and artifact removal | *Describe your procedure(s) for artifact and structured noise removal, specifying motion parameters, tissue signals and physiological signals (heart rate, respiration).*

Volume censoring | *Define your software and/or method and criteria for volume censoring, and state the extent of such censoring.*

Statistical modeling & inference

Model type and settings | *Specify type (mass univariate, multivariate, RSA, predictive, etc.) and describe essential details of the model at the first and second levels (e.g. fixed, random or mixed effects; drift or auto-correlation).*

Effect(s) tested | *Define precise effect in terms of the task or stimulus conditions instead of psychological concepts and indicate whether ANOVA or factorial designs were used.*

Specify type of analysis: ☐ Whole brain ☐ ROI-based ☐ Both

Statistic type for inference | *Specify voxel-wise or cluster-wise and report all relevant parameters for cluster-wise methods.*

(See Eklund et al. 2016)

Correction | *Describe the type of correction and how it is obtained for multiple comparisons (e.g. FWE, FDR, permutation or Monte Carlo).*

Models & analysis

n/a	Involved in the study
☐	☐ Functional and/or effective connectivity
☐	☐ Graph analysis
☐	☐ Multivariate modeling or predictive analysis

Functional and/or effective connectivity | *Report the measures of dependence used and the model details (e.g. Pearson correlation, partial correlation, mutual information).*

Graph analysis | *Report the dependent variable and connectivity measure, specifying weighted graph or binarized graph, subject- or group-level, and the global and/or node summaries used (e.g. clustering coefficient, efficiency, etc.).*

Multivariate modeling and predictive analysis | *Specify independent variables, features extraction and dimension reduction, model, training and evaluation metrics.*
